# Inhibition of *Streptococcus suis* Adhesion and Biofilm Formation *in Vitro* by Water Extracts of *Rhizoma Coptidis*

**DOI:** 10.3389/fphar.2018.00371

**Published:** 2018-04-16

**Authors:** Yan-Hua Li, Yong-Hui Zhou, Yong-Zhi Ren, Chang-Geng Xu, Xin Liu, Bing Liu, Jian-Qing Chen, Wen-Ya Ding, Yu-Lin Zhao, Yan-Bei Yang, Shuai Wang, Di Liu

**Affiliations:** ^1^College of Veterinary Medicine, Northeast Agricultural University, Harbin, China; ^2^Heilongjiang Key Laboratory for Animal Disease Control and Pharmaceutical Development, Harbin, China; ^3^Heilongjiang Academy of Agricultural Sciences, Harbin, China

**Keywords:** *S. suis*, biofilm, *Rhizoma Coptidis*, adhesion, real-time PCR, iTRAQ technology

## Abstract

*Streptococcus suis* is difficult to treat and responsible for various infections in humans and pigs. It can also form biofilms and induce persistent infections. *Rhizoma Coptidis* is a medicinal plant widely used in Traditional Chinese Medicine. Although the inhibitory effects of *Rhizoma Coptidis* on biofilm formation have been investigated in several studies, the ability of *Rhizoma Coptidis* to inhibit *S. suis* biofilm formation and the underlying mechanisms have not yet been reported. In this study, we showed that sub-minimal inhibitory concentrations (25 and 50 μg mL^-1^) of water extracts of *Rhizoma Coptidis* (*Coptis deltoidea* C.Y.Cheng & P.K.Hsiao, obtained from Sichuan Province) were sufficient to inhibit biofilm formation, as shown in the tissue culture plate (TCP) method and scanning electron microscopy. Real-time PCR and iTRAQ were used to measure gene and protein expression in *S. suis*. Sub-minimum inhibitory concentrations (25 and 50 μg mL^-1^) of *Rhizoma Coptidis* water extracts inhibited *S. suis* adhesion significantly in an anti-adherence assay. Some genes, such as *gapdh*, *sly*, and *mrp*, and proteins, such as antigen-like protein, CPS16V, and methyltransferase H, involved in adhesion were significantly modulated in cells treated with 50 μg mL^-1^ of *Rhizoma Coptidis* water extracts compared to untreated cells. The results from this study suggest that compounds in *Rhizoma Coptidis* water extracts play an important role in inhibiting adhesion of *S. suis* cells and, therefore, biofilm formation.

## Introduction

*Streptococcus suis* is a pathogen causing huge economic and financial losses in the pork industry and an emerging threat to human health (Staats et al., 1997; Hill et al., 2005; Lun et al., 2007). *S. suis* can form biofilms, trapping nutrients, and shielding the pathogen from antagonistic effects (Brady et al., 2008; Wang et al., 2011). Biofilms are consortia of microorganisms attached to biotic or abiotic surfaces. Generally, the initial step in biofilm formation is a non-specific, reversible attachment of bacteria to substrate surfaces. Once permanently attached, the bacteria start to synthesize insoluble exopolysaccharides that encase the adherent bacteria in a three-dimensional matrix (Costerton et al., 1987). Therefore, reducing *S. suis* adhesion to surfaces may be an effective way to mitigate biofilm formation.

Studies have suggested that some genes and proteins play crucial roles in a series of complex molecular processes leading to biofilm formation (Sauer, 2003; Latasa et al., 2006; Beloin et al., 2008; Gaddy and Actis, 2009). A previous study reported deletion of the *atl* gene from *S. suis* type 2 strain HA9801, which encodes an autolysin, reduced adhesion to HEp-2 cells by 50% compared with wild-type *S. suis*, suggesting a role for Alt in biofilm formation and cell adhesion (Ju et al., 2012). Glyceraldehyde 3-phosphate dehydrogenase (GAPDH), an *S. suis* protein, has been identified as an adhesin. GAPDH mediates cell adhesion, encouraging biofilm production (Brassard et al., 2004; Wang and Lu, 2007). Muramidase-released protein (MRP) is a cell wall protein allowing bacteria to resist phagocytosis by macrophages and aids adhesion to epithelial cells (Liang et al., 2011). MRP induces expression of the cell surface protein BapA1 in *Streptococcus pneumonia*. Deletion of *mrp* reduces the bacterium’s ability to aggregate and form biofilms (Liang et al., 2011).

*Rhizoma Coptidis* (RC), used for over 2000 years in Traditional Chinese Medicine, has been studied for its antibacterial, antiviral, anti-inflammatory, anti-hyperglycemic, and hypolipidemic effects (Ye et al., 2009; Wu et al., 2014). In recent years, there has been a surge in the study of plants rich in bioactive components. These components have been shown to possess various beneficial properties including anti-adhesive effects (Dixon, 2001). It is reported that *Rhizoma Coptidis* can inhibit biofilm formation by *Staphylococcus epidermidis* (Wang et al., 2009). Previous studies on the anti-pathogenic effects of *Rhizoma Coptidis* have focused on its anti-biofilm activity (Zhu and Li, 2006; Hayashi et al., 2007; Yu et al., 2007). Our previous study indicated that a water extract from *Rhizoma Coptidis* (*C. deltoidea* C.Y.Cheng & P.K.Hsiao, obtained from Sichuan Province), berberine hydrochloride and coptisine can all inhibit *S. suis* biofilm formation in a tissue culture plate (TCP) assay (Liu et al., 2015), though the mechanisms involved are poorly understood.

Since in the previous study we only found that water extract from *Rhizoma Coptidis* could interfere with the formation of *S. suis* biofilms, but we did not know the mechanism involved. So the aim of this study was therefore to investigate the mechanisms by which *Rhizoma Coptidis* (*C. deltoidea* C.Y.Cheng & P.K.Hsiao, obtained from Sichuan Province) extracts disrupt *S. suis* biofilm formation and bacterial adherence, and to guide strategies to prevent *S. suis* biofilm infection.

## Materials and Methods

### Preparation of Extract

*Rhizoma Coptidis* (*C. deltoidea* C.Y.Cheng & P.K.Hsiao, obtained from Sichuan Province) was purchased as a crude drug from the Beijing Tong Ren Tang Pharmacy. Its identity was authenticated by Professor Mingxia Bai at the Horticulture Branch of the Heilongjiang Academy of Agricultural Sciences. A berberine standard (877-200001) was purchased from the Ministry of Health of Drug Products. To generate water extracts of *Rhizoma Coptidis*, 50 g *Rhizoma Coptidis* powder were boiled in 500 mL distilled water for 60 min at 100°C before decanting and filtration. The filtrate was collected and added to 300 mL of distilled water and boiled for 60 min at 100°C. The final filtrate mass was lyophilized and concentrated into a dried powder with a yield of 0.25 g mL^-1^ and stored at 4°C. The amount of berberine, the major active ingredient, in *Rhizoma Coptidis* water extracts was measured by high-performance liquid chromatography (HPLC) on a Waters Alliance HPLC system (Waters e2695, United States) consisting of a binary pump and a UV/Vis detector. Separation was carried out using a 5 μm DL-Cl8 column (4.6 mm × 150 mm, Japan) at 37°C. Acetonitrile (solvent A) and 0.05 M potassium dihydrogen phosphate (solvent B) were used as the mobile phase at a ratio of 40:60 (solvent A:solvent B), supplemented with 0.015 M sodium dodecyl sulfate. The flow rate was set at 1.2 mL min^-1^. A detection wavelength of 345 nm and an injection volume of 5 μL were used in the study. The amount of berberine in the *Rhizoma Coptidis* aqueous extract was determined by comparing the HPLC retention time to the authentic standard. Quantification of berberine in the aqueous extract was done using a linear calibration plot of the peak area in HPLC at 345 nm against concentration using the external standard method. The calibration curve was calculated by plotting peak areas against six different concentrations of the standard solutions (0.1, 0.2, 0.4, 0.6, 0.8, and 1.0 mg mL^-1^).

### Minimum Inhibitory Concentrations

The MIC (minimal inhibitory concentration) was determined by the microtiter broth dilution method, as recommended by the Clinical and Laboratory Standards Institute [CLSI] (2016). Dilutions were performed in Todd-Hewitt Broth (THB) medium using 1 × 10^6^ colony-forming units (CFU) of bacteria per milliliter. Cell suspensions (100 μL) were inoculated into 96-well microtiter plates in the presence of *Rhizoma Coptidis* water extracts with different final concentrations (0, 1.625, 3.125, 6.25, 12.5, 25, 50, 100, or 200 μg mL^-1^). Azithromycin was used as a positive control, with the susceptibility (MIC) of *S. suis* ATCC 700794 to azithromycin found to be 32 μg mL^-1^ (Yang et al., 2016). Inoculated microplates were incubated at 37°C for 24 h before examination. Susceptibility (MIC) of *S. suis* ATCC700794 to *Rhizoma Coptidis* water extracts was 100 μg mL^-1^ (Berberine, the active ingredient, in *Rhizoma Coptidis* water extracts was 36.3 μg mL^-1^).

### Growth Conditions of *S. suis* Biofilms

*Streptococcus suis* ATCC700794 was grown overnight in THB (Sigma-Aldrich) at 37°C with constant shaking. Biofilm culture production was described previously (Wang et al., 2011). Briefly, *S. suis* grown in THB medium at 37°C was added to a 1% fibrinogen solution in 100 mm polystyrene dishes and grown for 24 h. After decanting the growth medium, plates were thoroughly rinsed twice with 50 mM Tris–HC1 (pH 7.5). Biofilms were then harvested by scraping. Cells were sonicated for 5 min and centrifuged at 12,000 × *g* for 10 min at 4°C. Supernatants were then removed and cell pellets were washed twice with 50 mM Tris–HC1 (pH 7.5).

### Determination of the Effect of *Rhizoma Coptidis* Water Extracts on Biofilm Formation by TCP Assay

*Streptococcus suis* cultures in mid-exponential growth phase with an optical density of 0.2 at 600 nm (OD_600_) were used for TCP assays. In each well of a 96-well plate, 100 μL of *S. suis* culture and 100 μL of *Rhizoma Coptidis* water extract were combined. The final tested concentrations were 6.25, 12.5, 25, or 50 μg mL^-1^. Wells filled with sterile growth medium were included as blank controls, azithromycin (1/2MIC) as a positive control. Wells containing 200 μL culture without extract served as negative controls. After incubation at 37°C for 24 h, all wells were washed with sterile phosphate-buffered saline (PBS) and stained with crystal violet.

### Scanning Electron Microscopy

Scanning electron microscopy (SEM) was performed as described previously (Zhao et al., 2015). Briefly, cultures were diluted to an OD_600_ of 0.1 before adding 2 mL to wells of a six-well microplate containing a 10 mm × 10 mm sterilized rough organic membrane (Mosutech Co., Ltd., Shanghai, China). After incubation without shaking for 24 h at 37°C, medium and planktonic bacteria were removed by washing with sterile PBS. Biofilms were then prepared for analysis (Zhao et al., 2015).

### Anti-adherence Activity of Extract Against *S. suis*

*Anti-adherence to organic membranes*. Assays were prepared as previously described (Hamada et al., 1981). Briefly, *S. suis* ATCC700794 cultures at mid-exponential growth phase were diluted to an OD_600_ of 0.1 before combining with 2 mL of THB or sub-MICs of *Rhizoma Coptidis* water extract in a six-well microplate containing a 10 mm × 10 mm sterilized rough organic membrane (Mosutech Co., Ltd., Shanghai, China). After incubation without shaking for 24 h at 37°C, planktonic cells were decanted. Attached cells were removed by addition of 0.5 M sodium hydroxide. Adherence was quantified by OD_600_. Percentage adherence = [OD_600_ of adhered cells/(OD_600_ of adhered cells + OD_600_ of planktonic cells)].

*Anti-adherence to cells*. Assays were prepared as described previously (Lalonde et al., 2000) with slight modifications. Briefly, PK-15 cells were cultured in DMEM (Hyclone) and grown in 75 cm × 75 cm flasks at 37°C with 5% CO_2_. Confluent monolayers of PK-15 cells (1.0 × 10^5^ cells per well) were cultured in 96-well plates (Corning, NY, United States). *S. suis* cells, either supplemented with sub-MICs of *Rhizoma Coptidis* water extracts or untreated, were added to each well at an MOI of 100:1 and incubated at 37°C to allow cells to attach. After 4 h, plates were washed twice with PBS and cells were lysed with sterile distilled water on ice. Both adherent and intracellular bacteria were counted on THB agar. Both assays were repeated three times.

### RNA Isolation and Real-Time PCR

Real-time PCR was performed as described previously (Yang et al., 2015). The primer sequences used in the experiment were shown in **Table 1**. To investigate the effect of *Rhizoma Coptidis* water extracts on gene expression, mid-log growth phase cultures of *S. suis* were supplemented with 50 μg mL^-1^ extract and incubated at 37°C for 24 h. Cells without extract served as control. Cultures were centrifuged at 10,000 × *g* for 5 min before treatment with an RNase Remover I (Huayueyang Ltd., Beijing, China). Total RNA levels were determined using the E.Z.N.A^TM^. Bacterial RNA isolation kit. Real-time PCR for each sample was performed as previously described (Yang et al., 2015).

**Table 1:** Primers used for the quantitative RT-PCR analysis.

Genes	Primer sequence
16S rRNA	Forward: 5′-TGCTAGTCACCGTAAGGCTAAG-3′ Reverse: 5′-GGCTGCAAGATTTCCTTGAT-3′
*gapdh*	Forward: 5′-GCTGAAGAAGTAAACGCTGCT-3′ Reverse: 5′-GTCGCATCAAACAATGAACC-3′
*sly*	Forward: 5′-AGTCAGTTTGGCACTCGTAGG-3′ Reverse: 5′-TTGTGGCTCGTAAGTCAAGC-3′
*mrp*	Forward: 5′-TGGCACAGTTATCAAGGAACC-3′ Reverse: 5′-TACCGTCAACACGAACCAAT-3′

### iTRAQ Analysis

Protein was extracted from *S. suis* cells either treated with 50 μg mL^-1^ *Rhizoma Coptidis* water extract or left untreated (Wang et al., 2011). iTRAQ analysis was performed at Shanghai Applied Protein Technology Co., Ltd. (APT, Shanghai, China). Three biological replicates were evaluated to minimize the influence of less reliable quantitative information. iTRAQ analysis was performed as previously described (Zhao et al., 2015).

### Statistical Analysis

Values were calculated as the mean of individual experiments in triplicate and compared to those of the control groups. Differences between two mean values were calculated by Student’s *t*-test using SPSS 11.0.0 statistical software, with *p-*values below 5% designated as statistically significant.

## Results

### Amounts of the Active Ingredient Berberine in *Rhizoma Coptidis* Water Extracts

**FIGURE 1:**
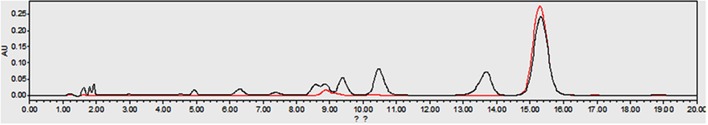
The HPLC chromatograms of the *Rhizoma Coptidis* aqueous extract (black, retention time 15.43 min) and the berberine standard (red, retention time 15.43 min).

High-performance liquid chromatography chromatograms of a *Rhizoma Coptidis* water extract and a standard solution of berberine are shown in **Figure 1**. The retention time of berberine agreed well with the authentic compound (15.43 min). The calibration curve equation was *y* = 7E + 06*x* + 28,724, *R*^2^ = 0.999. Using the calibration curve, the portion of berberine, the active ingredient, in *Rhizoma Coptidis* water extracts was calculated to be 36.30%.

### Effect of *Rhizoma Coptidis* Water Extracts Against Biofilm Formation *in Vitro*

**FIGURE 2:**
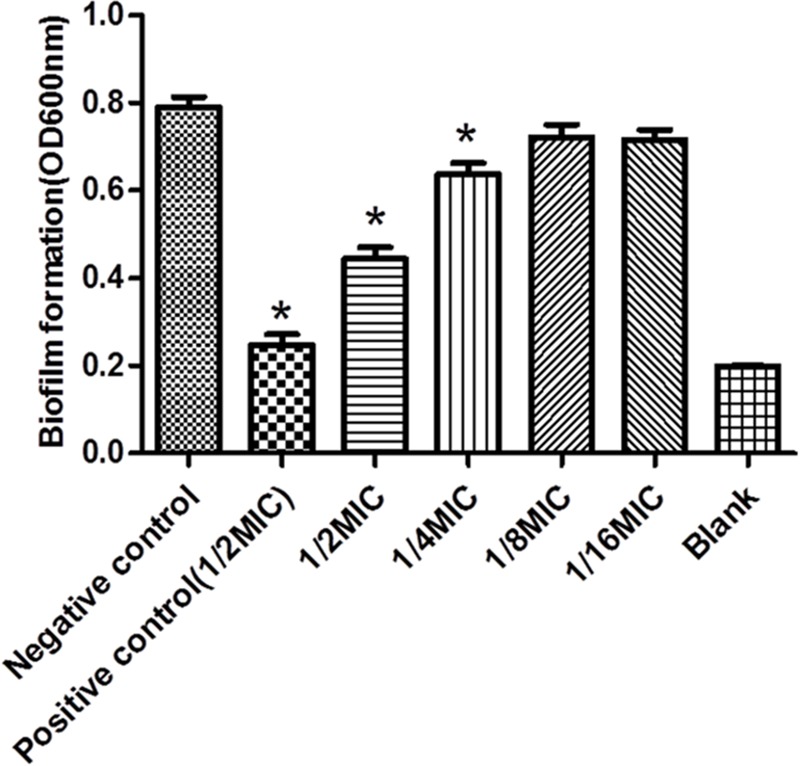
Effect of *Rhizoma Coptidis* at different concentrations on *S. suis* ATCC700794 biofilm formation. Data are expressed as means ± standard. Significant decrease (^∗^*p*<0.05) compared to control biofilm formation of *S. suis in vitro*.

The TCP method allows quantitative detection of *S. suis* biofilm formation at 24 h. Four different doses of *Rhizoma Coptidis* water extract were tested against *S. suis* biofilms (**Figure 2**). At 12.5 and 6.25 μg mL^-1^, the OD_600_ of *S. suis* ATCC700794 were lower than the negative control. At 25 and 50 μg mL^-1^, there was significant inhibition (*p* < 0.05) of *S. suis* biofilm formation, suggesting that these concentrations were more effective than 6.25 or 12.5 μg mL^-1^ at inhibiting biofilm formation.

### Scanning Electron Microscopy

**FIGURE 3:**
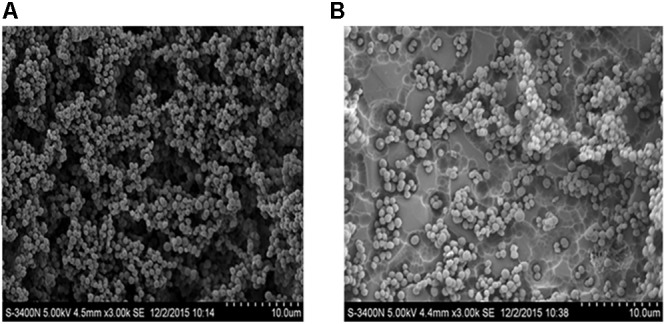
Scanning electron microscopy of biofilm of *S. suis* grown in THB broth. **(A)** With 0 MIC (0 μg mL^-1^) concentration of aqueous extracts of *Rhizoma Coptidis*; **(B)** with 1/2 × MIC (50 μg mL^-1^) concentration of aqueous extracts of *Rhizoma Coptidis*.

Scanning electron microscopy was performed to examine the effects of 50 μg mL^-1^ *Rhizoma Coptidis* water extract on *S. suis* biofilm formation. In the absence of extract, the surface of the rough organic membrane was observed to be almost entirely covered by aggregates and micro-colonies of *S. suis* (**Figure 3A**). However, when 50 μg mL^-1^ extract was added, most of the cell aggregates were dispersed (**Figure 3B**), suggesting that *S. suis* biofilm formation was inhibited by the extract *in vitro*.

## Anti-adherence Activity of Extract Against *S. suis*

**FIGURE 4:**
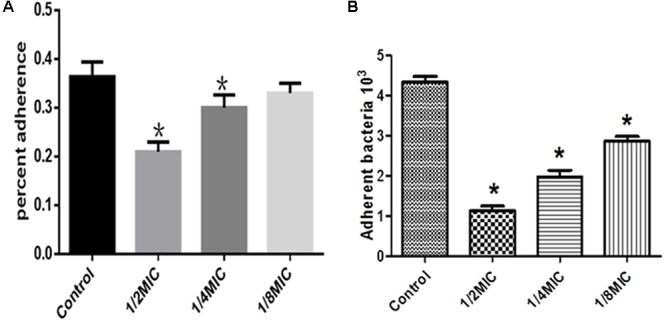
Effect of *Rhizoma Coptidis* at different concentrations on *S. suis* ATCC700794 adhesion to glass **(A)**, or PK-15 cells **(B)**. Data are expressed as means ± standard. Significant decrease (^∗^*p* < 0.05) compared to control *in vitro*.

The inhibitory effects of *Rhizoma Coptidis* water extract on adherence of *S. suis* to glass were tested at several concentrations (**Figure 4**). The extract inhibited adherence to organic membranes (**Figure 4A**) and PK-15 cells (**Figure 4B**).

## The Effect of *Rhizoma Coptidis* Water Extracts on Gene Expression

**FIGURE 5:**
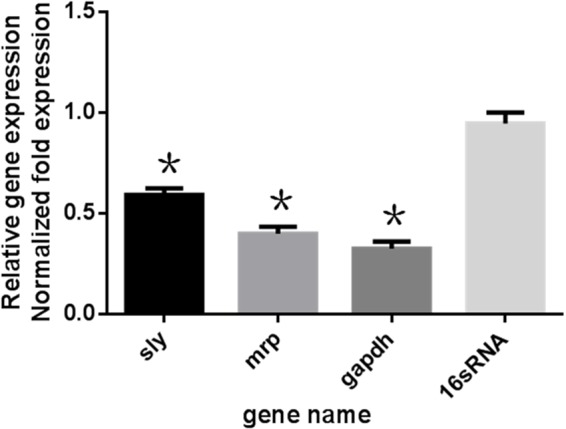
Effect of 1/2MIC of aqueous extracts of *Rhizoma Coptidis* on mRNA decreased expression of genes in *S. suis* ATCC700794. Data are expressed as means ± standard deviations. The expression was normalized to 16S rRNA. Controls refer to the absence of aqueous extracts of *Rhizoma Coptidis*. Significantly different (^∗^*p* < 0.05) compared to untreated control bacteria.

The expression profiles of *gapdh*, *sly*, and *mrp* in *S. suis* were determined 24 h post-treatment with 50 μg mL^-1^ *Rhizoma Coptidis* water extract. In treated cultures, *gapdh*, *sly*, and *mrp* gene expression levels were suppressed compared to untreated samples (**Figure 5**).

## *Rhizoma Coptidis* Water Extracts Inhibit Biofilm Formation and Modulate Protein Expression by iTRAQ

*Streptococcus suis* cultures were incubated with extract for 24 h before measurement using iTRAQ. Changes in protein expression levels were observed, with some proteins changing by more than 1.5-fold and others by less than 0.67-fold (*p* < 0.05). Of the 26 proteins tested using iTRAQ after treatment with 50 μg mL^-1^ extract, expression of 15 proteins increased and 11 were suppressed (**Table 2**). Among the suppressed proteins were antigen-like protein (D5AGH9), hydrolase (R4NST6), methyltransferase H (G7SM56), glycosyltransferase (M1VJJ3), and helicase (G7S7E3). These proteins had fold-change values of 0.495767886, 0.311630845, 0.644879525, 0.574502756, and 0.57612248, respectively.

**Table 2:** iTRAQ identification of differentially expressed proteins.

Accession	Proteins	Fold change
R4NST6	Hydrolase (HAD superfamily)	0.311630845
G7SER0	Putative uncharacterized protein	0.389353378
G7S2M0	DNA gyrase subunit B	0.427259889
D5AGH9	Antigen-like protein	0.495767886
G7SHZ3	Bacteriophage protein, putative	0.520090282
F4EDP5	Putative uncharacterized protein	0.534269172
G5KZN4	DNA polymerase IV	0.564882743
G7RZW0	Sugar ABC transporter permease	0.566616133
A4W3Y3	Response regulator	0.569410189
M1VJJ3	Glycosyltransferase	0.574502756
G7S7E3	Helicase	0.57612248
G7SM56	Methyltransferase H	0.644879525
R4NVK5	DNA gyrase subunit B	1.500773628
G7S2N4	ABC transporter ATP-binding protein	1.537200996
K0FG35	CpsR	1.542930507
R4NW55	AAA-class ATPase domain protein	1.55130354
G7SIQ5	Putative uncharacterized protein	1.568227687
F4EC05	Putative uncharacterized protein	1.724654275
E9NQ29	CPS16V	1.764868931
B9WUV5	Transcriptional regulator, DeoR family	1.913005357
B0FYB8	Neprilysin (Fragment)	2.203412347
G7SD52	ABC superfamily ATP binding cassette transporter, membrane protein	2.332354978
G7SM99	Type I site-specific restriction-modification system, R (Restriction) subunit and related helicase	2.805220661
G5L0Y1	ABC-type transport system involved in Fe-S cluster assembly, permease component	3.177157457
C6GT52	Chloramphenicol acetyltransferase	3.464547909
G7S7A9	FAD-dependent pyridine nucleotide-disulfide oxidoreductase	3.474123973

## Discussion

We investigated the relationship between *Rhizoma Coptidis* water extracts and *S. suis* biofilm formation. Previous studies have suggested that there is a relationship between some antimicrobial agents and biofilm formation (Majtan et al., 2008; Nucleo et al., 2009; Mishra et al., 2014; Zhao et al., 2015; Wang et al., 2016). In our study, sub-MICs of *Rhizoma Coptidis* water extracts could inhibit biofilm formation of *S. suis* *in vitro*, as observed in a TCP assay. Most studies on the anti-infective activities of *Rhizoma Coptidis* have focused on its anti-biofilm effects, with little or no attention paid to the specific mechanisms this effect. We detected anti-adherence activity of *Rhizoma Coptidis* water extracts against *S. suis*, suggesting that extracts of this plant inhibit adherence to organic membranes and PK-15 cells. Our results suggest that the anti-adherence activity of *Rhizoma Coptidis* water extracts is an important factor in inhibiting *S. suis* biofilm formation.

In this study, we identified *S. suis* genes *gapdh*, *sly*, and *mrp* as potential targets of *Rhizoma Coptidis* water extracts. These genes are thought to play key roles in infection and invasion (Liang et al., 2011; Ju et al., 2012) and have been shown to be important in biofilm formation and adhesion. Treatment with 50 μg mL^-1^ of extract suppressed *gapdh*, *sly*, and *mrp* gene expression. We speculate that this downregulation may be the cause of a reduction in *S. suis* adhesion and therefore biofilm formation. However, the detailed molecular mechanisms behind this reduction are still unknown and should be addressed in further studies.

Using iTRAQ, we found that 26 proteins were differentially expressed upon treatment of *S. suis* with 50 μg mL^-1^ *Rhizoma Coptidis* water extract compared to untreated cells. Of these proteins, 11 proteins, implicated in surface adhesion and biofilm formation, were significantly suppressed. These include an antigen-like protein (D5AGH9), hydrolase (R4NST6), methyltransferase H (G7SM56), glycosyltransferase (M1VJJ3), and helicase (G7S7E3) (**Table 2**). Antigen-like protein (D5AGH9) has been identified as a novel matricellular protein that promotes cell adhesion and spreading (Tajiri et al., 2010). Hydrolase, from the haloacid dehydrogenase superfamily (R4NST6), plays an important role in *Paracoccidioides brasiliensis* adherence to host cells (Hernandez et al., 2010). A previous study showed that deletion of an orphan C^5^-cytosine methyltransferase, similar to methyltransferase H (G7SM56), has a significant effect on the expression of genes responsible for pathogenic growth (Kumar et al., 2012). Over-expression of the putative *Brucella* glycosyltransferase can lead to development of clumping and increased adhesion to polystyrene plates (Dabral et al., 2015). Furthermore, a recent study showed that mutation of the *hrpB* gene, which encodes RNA helicase, can reduce surface adhesion and inhibit disease spread in citrus leaves (Granato et al., 2016).

Biofilm formation and adhesion were reduced by treatment with 50 μg mL^-1^ of *Rhizoma Coptidis* water extract, likely by downregulation of expression of the proteins discussed above. In contrast, loss of capsular polysaccharides has previously been described to facilitate and speed up biofilm formation (Qin et al., 2013). Our results suggest that treatment of *S. suis* cells by the extract might cause upregulation of CpsR (K0FG35) and CPS16V (E9NQ29), proteins involved capsular polysaccharide formation, and reduced biofilm formation and adhesion.

Our results show that sub-MICs of *Rhizoma Coptidis* water extracts could inhibit biofilm formation, though the mechanism of action is unclear. We observed anti-adherence activity of the extract on *S. suis*. We also found that expression levels of genes and proteins involved in adhesion were significantly altered in cells treated with sub-MICs of *Rhizoma Coptidis* water extracts compared to untreated cells. Our results indicate that *Rhizoma Coptidis* water extracts inhibit *S. suis* biofilm formation by limiting adhesion.

## Author Contributions

Y-HL designed the whole experiment. The other authors are responsible for completing the experiment.

## Conflict of Interest Statement

The authors declare that the research was conducted in the absence of any commercial or financial relationships that could be construed as a potential conflict of interest.
